# Sample Collection and Processing in Volatile Organic Compound Analysis for Gastrointestinal Cancers

**DOI:** 10.3390/diagnostics14141563

**Published:** 2024-07-19

**Authors:** Weiyang Zheng, Yiyang Min, Ke Pang, Dong Wu

**Affiliations:** 1 Department of Gastroenterology, Peking Union Medical College Hospital, Chinese Academy of Medical Sciences & Peking Union Medical College, Beijing 100730, China; 2 8-yr M.D. Program, Peking Union Medical College, Beijing 100730, China

**Keywords:** sample collection, volatile organic compounds, gastrointestinal tumor

## Abstract

Volatile organic compounds have drawn significant attention in recent years as a novel tool for non-invasive detection of a wide range of diseases, including gastrointestinal cancers, for which the need for effective, affordable, and non-invasive screening methods is substantial. Sample preparation is a fundamental step that greatly influences the quality of results and the feasibility of wide-range applications. This review summarizes sampling methods used in studies aiming at testing the diagnostic value of volatile organic compounds in gastrointestinal cancers, discussing in detail some of the recent advancements in automated sampling techniques. Finally, we propose some directions in which sample collection and processing can improve for VOC analysis to be popularized in clinical settings.

## 1. Introduction

**Figure 1:**
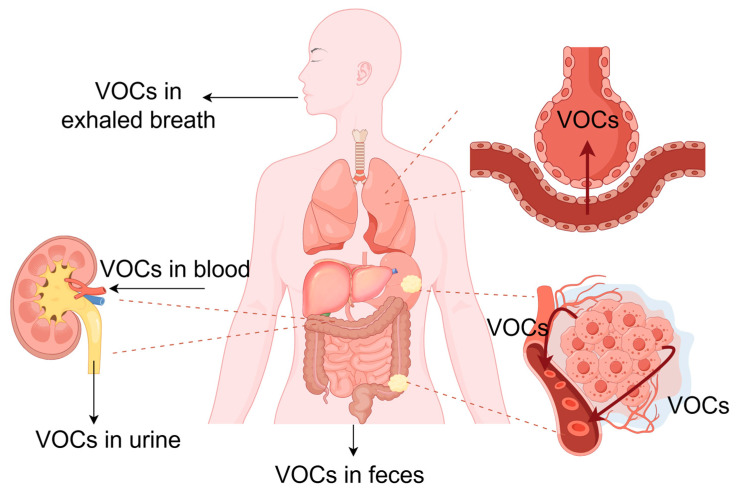
The distribution of volatile organic compounds from gastrointestinal tumor tissues to parts around the body. The cancer-related volatile organic compounds, produced as a result of metabolic alteration in cancer tissues, diffuse into the blood and are carried by the bloodstream to other parts of the body. They cross the alveolocapillary barrier and enter exhaled breath. By Figdraw (https://www.figdraw.com/static/index.html#/, accessed date: 9 July 2024).

Gastrointestinal cancers present a significant health burden, contributing to substantial morbidity and mortality rates worldwide. Gastric cancer (GC) and colorectal cancer (CRC) rank as the third and fourth leading causes of cancer death, respectively [1,2]. Apart from that, the physical, emotional, and socioeconomic consequences of cancer are profound, leading to decreased quality of life and financial hardships. Cancer screening is an important means for early diagnosis and intervention, being beneficial for reducing mortality and possibly reducing cancer incidence (by removal of precancerous lesions) [3]. CRC screening by sigmoidoscopy is estimated to be able to extend life for approximately 3 months [4]. Endoscopic approaches have the highest accuracy in screening and diagnosis of malignancies of the gastrointestinal tract [5,6]. However, being invasive and possessing a certain degree of risk, they are not recommended as a routine screening program except in regions with high prevalence [6,7]. There is still substantial need for non-invasive tests to screen for and help diagnose gastrointestinal cancers. Volatile organic compounds (VOCs) reflect the metabolic states and pathophysiological conditions of individuals [8,9] and have, in the recent decade, drawn attention as potential biomarkers to be utilized in screening for a variety of diseases, especially cancer. VOCs can be collected from breath, urine, feces, saliva, milk, skin secretions, blood, and tissues [10,11,12]. Taking into account the accessibility of sample sources and applicability to the general population, breath, urine, and feces have been most extensively studied. The distribution of VOCs from cancer tissues to other parts of the body, especially to these sample sources, is demonstrated in Figure 1. Though some attempts have been made to analyze blood and tissue VOCs for distinguishing cancer samples and healthy controls and yielded positive results [11,12,13,14,15], the primary application for these approaches is in research rather than clinical settings. Sample preparation is the first step of VOC analysis. The quality of samples is vital for the accuracy of results, and the ease of sample handling is an important factor to consider in the generalizability of an analyzing system. Various methods have been developed for the acquisition and preparation of samples, with no unified protocol or standard. This review focuses on sample preparation methods that have been developed in human VOC analyses, discusses their advantages and disadvantages, and emphasizes their applicability in medical practice. For reasons described above, this review will mainly discuss collection of breath, urinary, and fecal samples.

## 2. Volatile Organic Compounds as Biomarkers

Given the need for the early diagnosis of cancers, a variety of biomarkers have been explored. For CRC, currently, the most widely used biomarker is hemoglobin, detected by the fecal immunochemical test (FIT). This method is easy to conduct and demonstrates good sensitivity, but it does not guarantee satisfactory specificity, since gastrointestinal bleeding can occur under various non-cancerous circumstances. In a diagnostic accuracy study conducted in Britain from 2017 to 2019, when the sensitivity of FIT for CRC reached 97%, its specificity was only 64.9% [16]. Inadequate participation rate is another obstacle in applying FIT for CRC screening programs, which is probably due to patient reluctance in collecting fecal samples [17]. Other protein biomarkers for gastrointestinal cancers include carcinoembryonic antigen (CEA), CD26 protein (sCD26), CA 19-9, and so on. However, none of them display satisfactory sensitivity and specificity [17,18]. In a meta-analysis assessing different biomarkers for CRC, CEA used as a single marker only provided sensitivities ranging from 0 to 40% [17]. Some researchers focus on tumor-associated DNA and RNA as biomarkers, for example, circulating tumor DNA (ctDNA), DNA methylation markers, microRNA, and circular RNA. These fragments can be detected in the bloodstream and may carry distinct tumor-associated genetic and/or epigenetic characteristics. To this day, circulating tumor DNA (ctDNA) is mostly utilized as a prognostic marker, providing information about minimal residual disease (MRD), recurrence, and therapeutic response [19,20,21]. 

Volatile organic compounds first exhibited the potential of indicating cancers when various studies in the 1980s discovered that trained dogs can smell certain types of cancer [22]. But it was not until recent decades that significant advancements were made in analytical methods. In the context of cancer research, VOCs are mostly studied for the purpose of screening and early diagnosis. Nevertheless, there have been studies that investigate their value as prognostic markers. For example, Markar et al. found that among breath VOCs, propanal may serve as a marker of CRC recurrence, with a sensitivity of 71.4% and a specificity of 90.9% [23]. A pilot study conducted by Steenhuis et al. demonstrated that breath VOC analysis by electronic nose may be useful in detecting local recurrence or metastases of CRC [24]. Škapars et al. suggested that VOC analysis could be used for the surveillance of GC after surgery [25]. However, further prospective studies are needed to confirm the value of VOCs in this aspect.

Compared with the biomarkers mentioned above, which are mostly detected in blood, VOCs possess a unique advantage as they can be detected in completely non-invasive ways. Breath analysis is especially convenient and highly acceptable for patients. VOCs show promising abilities in the early diagnosis of gastrointestinal cancers better than that of current protein markers. The details will be described in the sections that follow. The substantial heterogeneity among existing studies hinders the application of VOCs as cancer biomarkers, but many other biomarkers face similar problems, such as microRNAs [18]. The detection of all the biomarkers mentioned above, except for hemoglobin in the feces, requires specialized equipment and staff. In addressing this matter, an electronic nose may be a distinctive and innovative solution. It is fast, lower in cost, and does not require specialized personnel. Detailed features of VOC analytical methods will be elaborated in the following text. 

## 3. Breath

**Figure 2:**
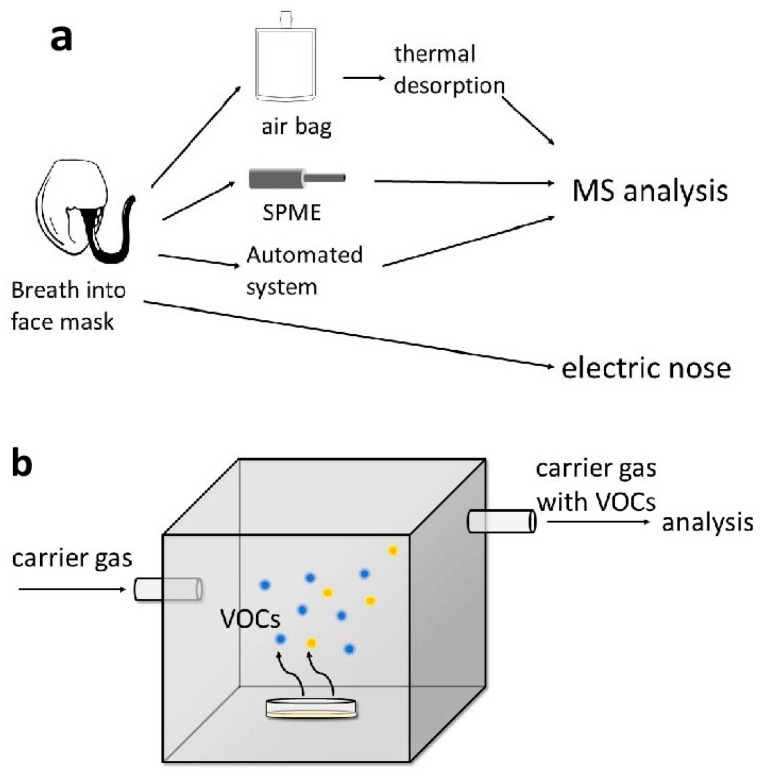
Collection procedure for different forms of samples. (**a**) Major methods of collecting breath samples and the corresponding analytical methods. (**b**) Headspace VOC collection for urinary and fecal samples.

Breath samples are easy to obtain and well accepted by patients, but they pose certain challenges in handling and storage. While the matrix is relatively simple, it can be influenced by factors such as oral environment. Miekisch et al. compared three modes of breath sampling and drew the conclusion that alveolar breath contains the least amount of exogenous products [26]. Due to this reason, alveolar breath has been preferred by many researchers as a subject for sample collection [27,28,29]. The collection of alveolar breath is achieved by monitoring CO_2_ concentration [26,27]. The major approaches in breath sample collection and analysis that have been tested for feasibility on human subjects are summarized in Table 1. A brief diagram of the collection procedure is demonstrated in Figure 2a. Traditionally, exhaled breath is collected in inert sample bags which exhibit no affinity or reactivity for any constituents in the gas. One commercially available and widely used product is the Tedlar^®^ bag. The bags serve as a medium for collection and storage. However, the concentration and recovery rate of VOCs in the Tedlar^®^ bag is significantly affected by temperature [30]. After collection into bags, sample air needs further processing before entering the analytical unit. The thermal desorption (TD) technique is central to the enrichment of VOCs prior to analysis [31,32,33]. In TD, VOCs that pass through the sorbent tubes are trapped on the sorbents and released under a certain higher temperature. The released VOCs, now enriched, can be carried to analyzing machines in a clean, inert air flow [34]. 

Solid phase microextraction (SPME) is a technique that overcomes some of the disadvantages of traditional sorbent tubes. The SPME device is composed of a holder and a fiber assembly. The coating extracting polymer can be guided into and out of a needle, which enables both long-term sampling (time-weighted average sampling, TWA) and grab sampling, the latter of which cannot be achieved by sorbent tubes. SPME combines extraction, preconcentration, and transfer of the sample into one device, thereby simplifying the process of sample collection. It allows for both air and liquid sample collection and also possesses the advantage of being reusable [53,54].

In recent years, automated breath sampling systems have also been developed and significantly simplified the process of breath collection, while providing easier control for confounding variables [55]. The ReCIVA^®^ system and the Mistral system are examples in this respect. The ReCIVA^®^ system, with built-in TD tubes and CO_2_ and pressure monitoring units, combines collection and enrichment in one apparatus and allows for the simultaneous collection of mixed breath and alveolar breath [55]. It has been applied in a number of studies on breath VOC analysis with optimistic results [14,56,57]. 

After the VOC collection procedures, the VOCs in the TD tubes are ready for analysis in the gas chromatography–mass spectrometry (GC-MS) system [35]. GC-MS is currently the gold standard for VOC analysis, due to its ability to identify the specific compound composition of one given sample. The distinctive breath VOCs identified by studies differ to a great extent. Even for studies using the same sample collecting equipment and analytical method, the overlap between them is quite limited. For example, Altomare et al. used ReCIVA^®^ for sampling and GC-MS for analysis and identified eight distinctive VOCs for CRC, including methyl-benzene, ethyl-benzene, and other compounds [37]. Woodfield et al., while using the same collection system and analytical method, identified 14 VOCs, the most significant of which is propyl propionate [38]. Tetradecane was the only VOC identified in both studies. Many factors may be responsible for this situation, such as diversity of population, susceptibility of breath samples to external disturbance, and difference in sampling procedures. Currently, there is no unified VOC profile for gastrointestinal cancers. It might not be feasible to detect early gastrointestinal malignancies with only one VOC, but rather, a panel of VOCs could be used. The association between specific VOCs and the underlying disease is also poorly understood. Further investigation in this aspect may help build more solid connections between VOCs and cancers, and therefore enhance their clinical value. 

A significant drawback of analyzing VOCs with GC-MS is that the instruments are typically large in size and not easily portable. The analytical process is time-consuming, precluding the attainment of immediate bedside results, and thereby restricting its application in medical practice. 

More recently, portable GC-MS instruments have been developed [58], aiming to expand the range of applications of this technique. However, Marcillo et al. compared portable GC-MS devices to one latest, most advanced benchtop GC-MS instrument in VOC analysis, and the portable devices showed poorer performance in sensitivity, reproducibility, and quality of mass spectra data (evaluated by their similarity to library spectra) [59]. This suggests that, currently, the portable GC-MS technique can still not replace stationary GC-MS, and this method still needs further development in order to achieve bedside application.

Several other techniques based on MS that employ soft-ionization methods and break VOCs into limited fragments [60] enable real-time analysis, such as selected ion flow tube mass spectrometry (SIFT-MS), proton transfer reaction mass spectrometry (PTR-MS), and single-photon ionization mass spectrometry (SPI-MS). A TD sampling system can be coupled to the MS system for sample collection [61]. These MS methods can also be combined with the time-of-flight (TOF) technique. SIFT-MS and PTR-MS measure the level of selected molecules rather than provide the overall profile of the sample [62]. SPI-MS enables comprehensive detection of VOCs and is particularly advantageous in detecting non-polar VOCs. However, its recognition of specific particles also depends on existing information about cancer VOC markers [44]. Thus, for application in population-based screening programs, the methods mentioned above all rely on a well-founded cancer VOC database. 

In 2022, Huang et al. applied a surface-enhanced Raman scattering (SERS) sensor to breath analysis, with a newly designed tubular shape to better capture and enrich gas molecules. SERS utilizes the difference in Raman response to discriminate analytes [63]. It obtains a chemical fingerprint, with the ability to detect certain molecules, such as aldehydes and ketones. TD is not needed in this method and collected gas can be directly pumped into the SERS sensor. SERS does not inherently provide real-time results, but it is more cost effective than GC-MS and SIFT-MS [49].

The diagnostic accuracy of the above-mentioned methods is listed in Table 1. ReCIVA^®^ coupled with GC-MS can achieve fair sensitivity and specificity at the same time. The diagnostic accuracy of SIFT-MS is generally lower than that of GC-MS. Profound heterogeneity exists among the studies and a comprehensive analysis is needed to evaluate the quality of breath VOC analysis with mass spectrometry technique. 

In the past decade, electric nose (e-nose) technology based on nanosensors and pattern recognition algorithms has been developed and gradually gained significant attention [64,65]. Sample breath is exhaled through a mouthpiece into a sensor chamber, which houses an array of nanosensors that can interact competitively with VOCs [51,66]. The output signal is a matrix of conductivity values that will be analyzed by machine learning models. Since VOC patterns in exhaled breath can be strongly affected by factors such as diet, smoking, alcohol intake, and ambient air, several measures are taken by researchers to minimize environmental noise. Carbon filters are used to eliminate contamination from the environment air. Additional high-efficiency particulate air (HEPA) filters can be used to block bacteria and viruses from entering the sensor chamber [51]. In some studies, patients are asked to go through fasting and refrain from smoking and alcohol drinking prior to the sample collection [50], while in some others, these factors are simply collected as baseline information [66]. Several studies have attempted to examine the influence of confounding factors, such as food ingestion [67], on the performance of e-noses, but there is not yet a clear conclusion. No standard has been established for sampling in e-nose analyses.

The diagnostic performance of e-nose for cancers has been systematically reviewed by Sheepers et al. A pooled analysis for CRC showed a sensitivity of 93% and a specificity of 59% [68]. It should not be neglected that the studies included generally show a high risk of bias, which is probably relevant with the factors discussed above. 

Simplifying the sampling procedure and applying nanosensor and machine learning technology, e-noses reduce the length of analysis to a great extent. The total analytical procedure (from the start of one breath to the readiness of the next) for Aeonose™, an e-nose product, only takes 15 min [24]. Despite its obvious advantages in operational simplicity, portability, rapid analysis, and cost-effectiveness, e-noses have not yet been employed in clinical settings for tumor diagnosis [68]. This is largely due to the high heterogeneity of results among different machines. The opacity of the analyzing process and underlying principles of machine learning techniques, one keystone technique in e-nose analysis, hamper the reproducibility of outcomes and the comparability of results produced by different machines, or even by the same machine but under different environmental conditions.

## 4. Urine

Analyzing VOCs using urinary samples has certain advantages. Urinary sample collection can be completed on-demand and is easily accepted by patients. It is relatively chemically stable, less likely to be affected by confounding factors such as diet, and can be stored long-term easily [69,70]. Urinary VOCs have been analyzed in attempts to detect cancers of the urinary tract, breast cancer, lung cancer, and others. Among neoplasms of the gastrointestinal system, its value in the detection of colorectal cancer has been most extensively studied. The analysis of urinary VOCs is most often based on headspace analysis. A schematic demonstration of headspace analysis is shown in Figure 2b. Field asymmetric ion mobility spectrometry (FAIMS) coupled with a standardized sampling system (for example, the ATLAS sampling system) is a method frequently used. Samples are heated to a designed temperature in the collection chamber. Carrier air flows through the headspace and into the FAIMS instrument, carrying VOCs from the sample [71,72]. VOCs from the headspace are ionized first and then pass through two parallel plates between which an alternating electric field is applied. The path of an ion is determined by the differential mobility that it shows in the high- and low-strength fields. Under a given dispersion field and compensatory voltage (one that compensates for the ion’s vertical drift and allows certain ions to pass through the plates), a certain type of ion is selected. By scanning through the fields, a complex pattern, containing information about the composition of the VOCs in the sample, can be obtained [70]. FAIMS does not identify specific compounds but rather produces a fingerprint. The clinical accuracy of FAIMS in differentiating people with and without CRC varies. Sensitivity can range from a little over 60% to 100%. Widlak et al. demonstrated that the sensitivity and specificity of FAIMS analysis of urinary VOCs can be improved when combined with FIT [73]. The diagnostic ability of urinary VOC analysis with the FAIMS method has not been comprehensively and systematically reviewed. 

More traditional GC-MS and GC-IMS can also be used for urine headspace analysis, though less often [72,74]. Urinary VOC identification faces the same difficulty as breath VOCs: no single VOC was consistently associated with cancer across studies. According to van Liere et al., currently, the most distinctive VOC for CRC may be butanal, a marker of oxidative stress [69].

McFarlane et al. used a sequence of liquid chromatography (LC), FAIMS, and MS to analyze urinary VOCs instead of direct headspace analysis. The sample, first eluted by LC, is aerosolized and sent into FAIMS, with MS following [75]. This allows for finer separation of different compounds and may increase the sensitivity and accuracy of VOC analysis. However, treating the sample in this sequence renders the analysis more time consuming and increases the complexity of the operation. Alterations also need to be made in data analysis, since data obtained by LC and FAIMS do not abide by a one-to-one corresponding relationship.

## 5. Feces

Fecal material has long been utilized as a sample source for non-invasive laboratory examination in the diagnosis of gastrointestinal diseases. Nevertheless, relatively fewer studies have been conducted in fecal VOC analysis when compared to urinary and breath VOCs. This might be due to the lower patient acceptance for fecal sample collection than for urine and breath. Moreover, fecal samples are heavily affected by exogenous factors such as diet and activity of the gut microbiota.

The sampling process of feces is generally similar to that of urine. Both sources are not in the gaseous phase, so analysis mainly focuses on the sample headspace. The techniques previously described, namely GC-MS and e-nose, can also be applied to fecal samples. SPME can also be applied to fecal headspace [76]. The micro-chamber/thermal extractor (μ-CTE) has been developed and employed as a sampling system for fecal VOCs in recent years. This portable device can carry out headspace VOC extraction in six chambers simultaneously, with temperature and carrier air flow rate customized according to software design. A variety of sorbent tubes can be connected to it and collect VOCs for further analysis, usually by GC-MS [77,78]. The diagnostic ability of fecal VOC GC-MS analysis seems promising. Śmiełowska et al. used an artificial neural network (ANN) to build a predictive model and achieved a diagnostic accuracy of 100% [78].

Alustiza et al. designed a headspace extraction unit based on magnetic adsorption using graphene oxide and Fe_3_O_4_ nanocomposite [79]. The underlying technique, known as magnetic solid-phase extraction (MSPE), combines a magnetic phase and a sorbent phase, thereby possessing both strong adsorption capacity and manipulability by magnetic forces [80]. The VOCs are extracted and transferred into a thermal desorption tube with the help of magnetic power. The tube can then be connected to a thermal desorption–gas chromatography–mass spectrometry (TD-GC-MS) system for analysis. 

As they rely on GC-MS for analysis, the above-mentioned devices cannot perform real-time analysis. These techniques are valuable for investigational purposes, since they can achieve high-quality sampling and recognition of specific VOC profiles but may not be suitable in clinical settings. 

Several studies have attempted to utilize e-noses in fecal VOC analysis. De Meij et al. used Cyranose 320^®^ to analyze fecal headspace. Frozen fecal samples are taken into a vacutainer and incubated at 37 °C to generate ample vapor which would pass into the e-nose through a connecting needle [81]. SCENT A1, a system based on nanostructured semiconductor gas sensors, has been tested and shown to have better positive predictive value than FIT in distinguishing healthy people and individuals affected by colorectal tumors [82]. It contains a pneumatic system that directs filtered air through a sample box, transporting fecal exhalation into the sensor unit. Fecal samples are frozen at −8 °C and need to be defrosted 30–40 min before the analysis. The whole process of one analysis takes around 50–70 min, with 20–40 min for analysis and 30 min for self-cleaning to prepare for the next analysis [83,84]. These two e-nose devices showed similar diagnostic power, with sensitivities and specificities around 85%. It should be noted, however, that existing studies on fecal VOCs, regardless of the analytical method used, are small in both size and number. Further validation is needed to acknowledge the diagnostic power of fecal VOC analysis.

A different approach taken by Ishibe et al. exploited defecation gas components instead of feces. Their sampling system is placed in a toilet and consists of a fan that aspirates air, a gas sensor, a pipe that connects the gas sensor and the sampling Tedlar^®^ bag, and a stopper placed in front of the bag, the opening of which is controlled by the gas sensor. When the toilet is in use, the system collects the defecation gas of patients for 30 s before the stopper automatically closes. Toilet air is aspirated when the toilet is not in use and treated as background [85]. For subsequent analysis, gas in the sample bag is injected into a GC system. This study demonstrated that the defecation gas of CRC patients contains significantly higher concentrations of methyl mercaptan but did not provide information about the predictive value of this method. This method is highly patient-friendly, as it requires no additional actions beyond routine daily activities from the patients. Combining this sampling apparatus with e-noses may obviate the requirement for GC-MS analysis and enable more affordable real-time analysis. However, the feasibility of the regular installation and maintenance of this equipment in hospitals remains a problem.

## 6. Conclusions

Overall, among the VOC sample sources, breath is probably the earliest to be investigated and most extensively studied. Compared with mixed breath, alveolar air is less affected by exogenous components and more stable than mixed air or time-controlled samples. Equipment used for breath collection includes inert bags, sorbent tubes, SPME tubes, and automated sampling systems. Samples collected in bags need to be transferred onto sorbent tubes before analysis for VOC enrichment. The ReCIVA^®^ system is one commercially available apparatus that is used relatively often for automated breath collection and processing. It allows for the simultaneous collection of mixed breath and alveolar breath. E-nose technology that enables on-line analysis does not require extra maneuvering of the sample. Breath is filtered to minimize the contamination of samples and passes directly onto nanosensors that generate a fingerprint of the input gas.

For urine and feces, the mainstream method of sample collection utilizes their headspace. In urinary VOC analysis, one of the most often employed techniques is FAIMS coupled with a standardized sampling system. The Owlstone ATLAS sampling system enables air-flow control and temperature control. Clean compressed air carries headspace VOCs into the analyzer for on-line analysis. For fecal samples, instruments such as the μ-CTE (for GC-MS) and SCENT A1 (for e-nose) have been developed. 

In summary, there are mainly two routes for VOC sample preparation. For off-line analysis that can distinguish specific VOC components, samples need to be enriched and stored before analysis, meaning that the sample preparation process usually involves more steps. The TD technique is central to the enrichment process. For on-line analysis that recognizes sample VOC patterns but not their specific composition, the sample does not need pre-concentration, but the control of sampling condition and elimination of distractors are important for data quality. The limitations of the former method are that it is usually time-consuming and requires more professional manipulation. There is also the problem of equipment cost and transportability. The latter method complements these disadvantages excellently, but it faces many challenges such as the instability of data and lack of comparability. The difficulty in establishing a unified standard impedes the implementation of these techniques under clinical scenarios. Despite the fact that, based on existing data, VOCs appear to be promising biomarkers for the early diagnosis of gastrointestinal cancers, these technical barriers still need to be overcome for VOC analyzing systems to be implemented for routine hospital use.

For future development of VOC analysis for screening and diagnosing gastrointestinal cancers, advancements in both sample collection and analytical techniques are equally crucial. Automated sampling systems represent a significant advancement in the field of sample collection and may contribute to achieving better consistency across different settings. Optimization of sampling conditions and standardization of sampling procedures may be helpful for improving the external validity of VOC analysis results.

## Figures and Tables

**Table 1:** Characteristics of different approaches in breath sample collection and analysis.

Method of Collection	Method of Analysis	Real-Time Analysis	Identification of Individual Compounds	Duration of Procedure	Target	Sensitivity/%	Specificity/%	References
Tedlar^®^ bag, TD	GC-MS	no	yes	~2 h	CRC	96	83	[35]
Visually CO_2_-controlled sampling, SPME	GC-MS			-	CRC	-	-	[28]
SPME	GC-MS			-	GC	-	-	[36]
ReCIVA^®^, TD	GC-MS			-	CRC	79–90	86–93	[37,38]
ReCIVA^®^, TD	Multi-MS ^a^	no	yes	-	Esophageal-gastric cancer	-	-	[39]
Nalophan bag	SIFT-MS	yes	selected	-	CRC	96	76	[23]
Nalophan bag	SIFT-MS			-	Esophageal and gastric adenocarcinoma	86.7	81.2	[40]
Steel breath bag	SIFT-MS			-	Esophagogastric cancer	80	81	[41]
Mylar^®^ bag	SIFT-MS				Hepatocellular cancer	73	71	[42]
Tedlar^®^ bag, TD	PTR-TOF-MS	yes	selected	-	GC	61	94	[43]
Tedlar^®^ bag, TD	SPI-MS	yes	partly	-	GC	95.8	96.5	[44]
BioVOC™, Tedlar^®^ bag	UVP-TOFMS ^b^	yes	partly	-	Upper gastrointestinal cancer	92.3	100	[45]
Mylar^®^ bag	nanoarray	yes	no	-	CRC	85	94	[29]
Mylar^®^ bag	nanoarray			-	Precancerous gastric lesions and gastric carcinoma	73	98	[46]
BioVOC™	nanoarray			15 min for sampler 20 min for sensor	GC	100	93	[47]
Tenax^®^ TA sorption tube	nanoarray			-	GC	100	98	[48]
Tedlar^®^ bag	SERS	no	partly	-	GC	91.23	88.52	[49]
e-nose		yes	no	-	CRC	63.3	84.2	[50]
Aeonose™				15 min	CRC	95	64	[51]
Aeonose™					GC	81	71	[52]

^a^ This MS platform incorporates GC-MS retrofitted with electron ionization (EI) and positive chemical ionization (PCI) and SIFT-MS. ^b^ UVP-TOFMS: ultraviolet photoionization time-of-flight mass spectrometry, another kind of soft ionization technique.
